# The Works of Dr. Hall

**Published:** 1877-06

**Authors:** 


### *THE WORKS OF W. W. HALL.*

The writings of Dr. Hall that have given him his great reputation are comprised in the volumes of the JOURNAL OF HEALTH during the twenty years that he was its editor. Whatever he published outside of the JOURNAL during that twenty years, or whatever has been published since, drew its inspiration from the pages of the JOURNAL, which was his real "life work." We have but a few hundred copies of the bound volumes left, and when they are sold the books will be out of print, probably never to be re-published. One bound volume of Hall's JOURNAL OF HEALTH, containing 250 pages, will be sent post paid to any address, on receipt of 60 cents. One bound volume 500 pages, $1.00. Two bound volumes, 250 pages each, and one of 500 pages, post-paid, $2. We will also send either of the following works of Dr. Hall, post-paid on receipt of $1.50: "How to Live Long," "Health by Good Living," "Sleep; or the Hygiene of the Night," "Health and Disease as Affected by Constipation," "Bronchitis and Kindred Diseases," "Coughs and Colds," "Dyspepsia." The work on "Dyspepsia" is published by R. Worthington. It was Dr. Hall's last work. He had just completed it, but had not corrected the manuscript when he died. It is certainly up to the standard of his other writings, and bears the evidence of his clear and vigorous style. Although but recently published, it is meeting with a large and increasing demand. Dr. Hall's "Guide Book," or "Family Doctor" will be sent free by mail on receipt of $4.50. Address HALL'S JOURNAL OF HEALTH, New York. All letters on professional business should be addressed to Dr. E. H. Gibbs, Editor of HALL'S JOURNAL OF HEALTH, New York.

